# The Relationship between Signs of Medical Conditions and Cognitive Decline in Senior Dogs

**DOI:** 10.3390/ani13132203

**Published:** 2023-07-05

**Authors:** Rosalind Wrightson, Mariangela Albertini, Federica Pirrone, Kevin McPeake, Patrizia Piotti

**Affiliations:** 1 The Royal (Dick) School of Veterinary Studies, Midlothian, Edinburgh EH25 9RG, UK; wrightbehaviour@outlook.com (R.W.); kevin.mcpeake@ed.ac.uk (K.M.); 2 Department of Veterinary Medicine and Animal Science, University of Milan, 26900 Lodi, Italy; federica.pirrone@unimi.it (F.P.); patrizia.piotti1@unimi.it (P.P.)

**Keywords:** canine cognitive dysfunction syndrome, CDS, cognitive assessment scale, dog, ageing

## Abstract

**Simple Summary:**

As dogs age, they develop conditions associated with old age, which include pathologies of the brain and the body. One of these conditions is canine cognitive dysfunction syndrome, a neurodegenerative disorder similar to the early stages of human Alzheimer’s disease. The goal of the current study was to investigate the relationship between cognitive dysfunction syndrome and other medical conditions in geriatric dogs, as reported by their owners, using an online questionnaire. The results indicated that dogs with greater cognitive dysfunction also had more signs associated with musculoskeletal and neurological problems, including pain and sensory decline. The relationship was similar, but weaker, for symptoms of digestive or metabolic disorders, and it was weakest for dermatological symptoms. The take-home message of the current study is that, in older patients, medical conditions should be carefully screened, and in cases of suspected cognitive dysfunction, the underlying cause should be cautiously assessed, especially for pain, sensory decline, and digestive and metabolic conditions.

**Abstract:**

Canine cognitive dysfunction syndrome (CCDS) is a progressive age-related neurodegenerative disorder in dogs. Minimal research has been performed to investigate how clinical signs may be impacted by other medical conditions. A cross-sectional study was performed using the Canine Cognitive Assessment Scale (CCAS) to evaluate cognitive impairment as reported by owners. Owner-reported health-related measures included behaviour changes, the body condition score, and veterinary diagnoses of disease. The responses from 804 dogs in the last 25% of their expected lifespan were analysed. Factors were identified in the owner-reported behavioural signs of disease representing pathologies in four body systems: musculoskeletal–neurological, digestive, metabolic, and dermatological, with the items comprising these factors also compiled into a cumulative measure of health. The results showed a strong correlation between the CCAS score and both the musculoskeletal–neurological factor and the overall cumulative measure of health. Moderate correlations between the CCAS score and the digestive factor and metabolic factor were also observed. The correlation between the dermatological factor and the CCAS score was weak. This study highlights the need to screen dogs for concurrent diseases when using scales to assess cognitive impairment and to monitor dogs who have health conditions, particularly those that are painful, for the onset of cognitive impairment.

## 1. Introduction

Canine cognitive dysfunction syndrome (CCDS) describes an age-related disorder featuring cognitive impairment, which is analogous to the early stages of Alzheimer’s disease (AD) in humans [1]. The prevalence of cognitive impairment in older dogs, as reported in the literature, varies widely, with the comparison between studies being hampered by the different scales used and populations studied. The published figures range from 14.2% in dogs over 10 years of age [2] to 22.5% in dogs over 9 years of age [3] to as high as 74% in dogs over 7 years of age [4]. Age is a major risk factor for the increasing prevalence and severity of CCDS [2,3,5]. The authors observed that over a period of 24 months, 33% of dogs over eight years of age with normal cognitive function progressed to mild cognitive impairment (MCI), and 22% with MCI progressed to severe cognitive impairment [6]. A similar pattern of findings was reported by Madari et al. [7]. The true prevalence of CCDS in the canine population may be even higher than it was estimated to be due to under-diagnosis, with a discrepancy reported between cognitive impairment as measured using a scale, 14.2%, and that diagnosed by a veterinarian, 1.9%, in a population of dogs over 10 years of age [8]. This was, in part, attributed to under-reporting by owners, who may consider the behavioural changes to be a part of normal ageing [9].

CCDS is also important due to its impact on the well-being of the dogs’ human guardians. The caregiver burden is the negative impact on a caregiver of having to care for an individual with a chronic condition [10], which, in veterinary medicine, is linked to making a decision about euthanasia [11]. The caregiver burden associated with owning a dog with CCDS has not been investigated directly; however, it is likely to be high due to owners having to alter their routines, accommodate the dog’s changing requirements, and the impact of the behavioural symptoms associated with CCDS [12]. Increasing age alone is a risk factor for euthanasia, while behavioural changes are the fourth most commonly reported reason for canine euthanasia [13], reflecting the negative impact of unwanted behaviours on the human–animal bond.

The diagnosis of CCDS relies on the identification of suggestive behavioural symptoms and the exclusion of other possible causes. Magnetic resonance imaging (MRI) allows the assessment of some gross intracranial changes that may be associated with CCDS, including cortical atrophy [14], leukoaraiosis (periventricular changes to white matter) [15], and the reduced size of the interthalamic adhesion [16]; however, these abnormalities are not specific to CCDS and are also found in senior and geriatric dogs without cognitive impairment [1]. MRI does serve a role in the exclusion of other intracranial pathologies, such as space-occupying lesions and cerebellar infarction [17]; however, the benefit of MRI often does not outweigh the cost and risks associated with general anaesthesia [1]. Authors have demonstrated the relationship between the accumulation of beta-amyloid plaques and CCDS’ cognitive deficit [18,19]. However, there is not yet sufficient evidence that beta-amyloid or other markers, such as TAU protein or Neurofilament Light Chain (NFL), could be used as markers of cognitive decline in dogs [20]. Due to the absence of disease-specific changes, an ante-mortem presumptive diagnosis relies on the identification of behavioural changes. An acronym, DISHA, is used to describe areas of behaviour that might be impacted, standing for disorientation, interaction, sleep–wake cycle, house soiling, and activity levels [21], and has gone through several iterations, with the addition of AAL (activity-apathy and depression; anxiety; learning and memory) [22]. These behavioural changes are non-specific, and so may be displayed as a result of many different pathological processes, including sensory decline and musculoskeletal disease [23,24], making CCDS a diagnosis of exclusion. To aid veterinarians in identifying these behaviours and the extent to which they are displayed, several scales have been developed, with some also allowing the categorisation of the degree of cognitive impairment [2,7,25]. Researchers are also trying to develop behavioural tests to assess the cognitive state of pet dogs as they age, with the aim of aiding the diagnosis of CCDS [26,27,28,29]. However, these still need further validation [25]. It is notable that although the scales may indicate the presence of cognitive impairment, it is still incumbent on the veterinarian to rule out other possible causes of the behavioural changes reported.

As an organism ages, the risk factors for disease increase, and the likelihood of multimorbidities rises [30]. As a result, there is often a complex clinical picture with the presence of behavioural signs that might be suggestive of CCDS accompanied by other physical disease states. This interplay between physical and cognitive health was the focus of the current study, considering how cognitive impairment, measured by a CCDS assessment scale, related to the presence of other disease states. This study aimed to understand: (1) how different disease states correlate with the degree of cognitive impairment in dogs; (2) what relationship exists between a cumulative measure of health and the degree of cognitive impairment in dogs. It was anticipated that there would be common pathological processes linking the different behavioural signs of the disease assessed and that these would be associated with greater cognitive impairment. It was hypothesised that greater cognitive impairment would also be associated with worsening overall health and with the presence of specific disease states.

## 2. Materials and Methods

### 2.1. Subjects

A cross-sectional study design was used to assess the relationship between owner-reported behavioural signs of disease and veterinary diagnoses, with canine cognitive impairment as indicated by the Canine Cognitive Assessment Scale (CCAS) [25]. The CCAS was selected due to the ability to categorise the degree of cognitive impairment based on resulting scores, as well as its inclusion of a broad range of behavioural changes, including behaviours relating to anxiety.

A convenience sample of participants was taken via social media platforms. The online questionnaire was active between 4 February 2021 and 22 March 2021. Participants needed to be over 18 years of age. To be eligible, they were also required to have owned their dog for over one year, in order for the participant to have knowledge of the veterinary diagnoses made during that period. The dogs needed to be over five years of age to allow the inclusion of dogs estimated to be in the last 25% of their estimated lifespan, which represents the senior age bracket in dogs [31]. This relatively young age was selected to capture data from large breed dogs that have a shorter estimated lifespan than smaller dogs [32,33]. If the participant owned more than one dog, they were asked to complete the survey considering their oldest dog.

### 2.2. Questionnaire

Participants were invited to complete an anonymous online survey about ageing in dogs hosted on the JISC Online Surveys platform. The survey comprised three sections:

(1) Demographic information about the dog—breed, weight, height, age, body condition score (BCS), and how recently it had been examined by a veterinarian;
(2) Assessment of the dog’s cognitive health—CCAS scale [25] comprising 17 items which assessed six domains of behaviour (disorientation, sleep–wake cycles, social interactions, learning and memory, activity level, and anxiety). The scale utilises a four-point Likert scale; never (0), once a month (1), once a week (2), almost every day (3), reporting behaviour over the previous six months. If a participant was unsure how to respond, they were asked to select ‘Never’ as opposed to leaving it blank as directed by the original CCAS;
(3) An assessment of the subject’s general health—37 questions regarding behaviours that reflect the pathology of different body systems, hereafter referred to as ‘general health questions (GHQs),’ and 12 questions regarding diagnoses made by a veterinarian in the preceding year, hereafter referred to as ‘diagnoses questions’ (see Supplementary Information). Questionnaires published in the literature concerning the assessment of health in older dogs [34,35,36] were used as a basis for the GHQs. Items were phrased in colloquial English to ensure that they were not ambiguous. The GHQs utilised a four-point Likert scale describing the degree of change. The diagnoses questions had binary yes/no response options.

### 2.3. Data Analysis

Statistical analyses were performed on IBM SPSS version 25. The weight and height of the dogs were used to calculate their expected lifespan, in order to account for the fact that dogs of different sizes age at different speeds [34]. The height and body weight of the dogs were used to calculate the estimated lifespan and hence select the data to use for further analysis using the formula [33] below, adjusted from imperial to metric units:

Estimated lifespan = 13.620 + (0.0276 H) − (0.1186 W)(1)

where H indicates the height of the withers (cm), and W indicates the body weight (kg).

The formula provides the theoretical lifespan (in years) for a dog based on its size. We then compared this value with the actual age of the dog to select those that had reached or were above 75% of their lifespan (the marker for senior age, according to the AAHA guidelines for senior dogs and cats).

As the height was collected as categorical information, the midpoint of each range was used to represent the height of the subject (for example, the range 21–40 cm was represented by 31 cm). In addition, two subjects were excluded from the analyses as the participant’s responses to the weight change questions were inconsistent (both weight gain and loss). Therefore, the analysis was run on 802 dogs. The overall CCAS score was calculated by summing single-item scores. In addition, the domain ‘disorientation’ was weighted as double. The final CCAS score indicated degrees of cognitive impairment ranging from normal ageing (scores 0–7), mild (scores 8–40), and severe (scores 41–69). Given that score data was used, non-parametric analyses were employed.

A Spearman’s rank order correlation test was used to measure the correlation between the CCAS scores and both the OHS and mean factor scores. Additionally, a Spearman correlation was also used to assess the relationship between subject weight and age with the overall CCAS score. The strength of correlation was assessed as absent (0–0.09), weak (0.1–0.29), moderate (0.3–0.49), or strong (0.5–1.0). A Kruskal–Wallis test was conducted to test whether there was a difference in the factor means between CCAS categories (normal ageing, mild cognitive impairment, severe cognitive impairment).

Dogs who had received a veterinary examination in the preceding year, n = 710, were included in a Pearson’s chi-square test to analyse the relationship between the CCAS categories and the conditions comprising the ‘Diagnoses’ variable. All expected cell frequencies were required to be over 1, and at least 80% greater than 5. When these criteria were not met, the categories of mild and severe cognitive impairment were merged, resulting in a two-category variable, ‘Presence of cognitive impairment’. The Pearson’s chi-square test was also utilised to assess the relationship between the BCS of the subjects and the presence of cognitive impairment. The categories of BCS 1–2 and 3–4 were merged to give an ‘underweight’ category, while BCS 6–7 and 8–9 were merged, giving an ‘overweight’ category. The strength of association was determined by calculating Cramer’s V, with interpretation adapted from Cohen [37]; small 0.1–<0.3, medium 0.3–<0.5, large ≥0.5.

Finally, we ran a cumulative link regression model with the CCAS score as the dependent variable and the four factors and age as fixed factors without interactions, in order to assess the combined effect of the medical signs and age.

The significance level was set at *p* < 0.05 for all tests. However, when multiple tests were performed, increasing the risk of type 1 errors [38], a Bonferroni correction [39] was applied (0.05/25 = 0.002), resulting in a significance level of *p* < 0.002.

## 3. Results

Data regarding 1374 dogs were collected. Of these, 804 dogs were calculated to be in the last 25% of their lifespan based on body weight and height, and their data were further analysed. The data set included a broad range of breeds, with over 74 named breeds making up 67.7% of the total (n = 544) and mixed breeds forming 32.3% (n = 260). The median age was 12 years (range, 5–19 years), the median bodyweight was 22 kg (range, 2–99), and the median height was 51 cm (range, 10–91). The body condition score (BCS) of the subjects, using a 1–9 scale [40], had a mode of BCS 5.

In this population, about half of the dogs had a CCAS score equal to normal ageing (n = 446, 55% of the population), 43% presented mild cognitive impairment (n = 345), and 2% had a score of severe cognitive impairment (n = 13). Dogs with cognitive impairment represented 45% of the population (n = 358).

**Figure 1:**
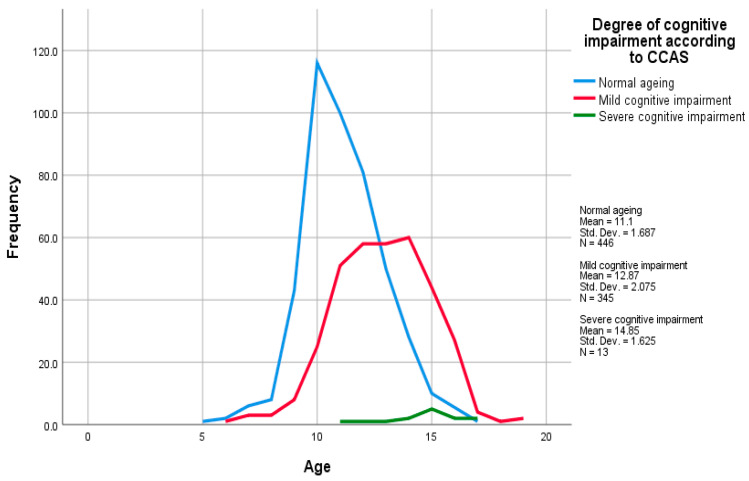
Multiple line graph of age by the degree of cognitive impairment according to CCAS score. The figure illustrates how the frequency of the different categories of cognitive impairment indicated by the CCAS score varied with age.

There was a negative but weak (r = 0.1–0.29) correlation between the CCAS score and bodyweight (Spearman’s rho, *rs* = −0.210, *p* < 0.001) and a positive, strong (r = 0.5–1.0) correlation with age (Spearman’s rho, *rs* = 0.507, *p* < 0.001), Figure 1.

It was observed that there was a statistically significant relationship between the presence of cognitive impairment (normal ageing and cognitive impairment) and body condition. Specifically, dogs with ideal weight were more common in the normal ageing group (underweight n = 114, ideal weight n = 493, and overweight n = 197), χ^2^ (2) = 21.251, *p* < 0.001. However, this was a small effect (Cramer’s V = 0.163, *p* < 0.001). All expected cell frequencies were greater than 5. There was no significant association detected between breed category (pure breed and mixed breed) and CCAS category (normal ageing, mild cognitive impairment, and severe cognitive impairment), χ^2^ (2) = 0.537, *p* = 0.764. All expected cell frequencies were greater than 1, with 16.7% of cells less than 5.

The GHQ items were assessed before performing the factor analysis. Eight GHQ items (avoids touch, assistance eating, constipation, flinches when eating, urinary incontinence, ear problems, alopecia, syncope) were excluded from the factor analysis due to exhibiting low variance determined by a standard deviation of less than 0.5 or greater than 90% of the dogs not exhibiting a score above 0. Four additional GHQ items (head pressing, circling, seizures, head tilt) with low variance were nevertheless retained as they related to neurological dysfunction and were considered clinically relevant by the authors. A principal axis factor analysis with promax rotation was performed on the remaining 28 items. In this, the minimum amount of data for factor analysis was satisfied, with a ratio of over 20 subjects per item [41]. Several criteria for the factorability of a correlation were used: that the determinant of the correlation matrix was greater than 0.00001; that the Kaiser-Meyer-Olkin (KMO) measure of sampling adequacy was 0.85 which falls into the ‘meritorious’ category [42]; that the KMO for all the individual items was greater than 0.52; and that Bartlett’s test of sphericity was significant (*p* < 0.001). The correlation matrix was visually checked, and none of the items had *r* > 0.9 with one another, thus excluding multicollinearity. Given these indicators, factor analysis was deemed to be suitable for all 28 items. The final correlation matrix can be viewed in Supplementary Information Table S1. An exploratory factor analysis was performed on the GHQ items to identify factor groupings within the data. The scree plot and eigenvalues were considered and used to determine how many factors to extract. Eight factors had an eigenvalue greater than 1; visual inspection of the scree plot revealed points of inflexion at two and three factors. Solutions for four and three factors were each examined, using varimax, oblimin, and promax rotations of the factor loading matrix. The four factors solution was preferred as it met the interpretability criterion, and promax rotation provided the best-defined factor structure. Thereafter factor extraction was an iterative process based on the examination of the pattern matrix, excluding variables that did not load above the cut-off of 0.4. The analysis was repeated until all remaining variables met the criterion level, with the four factors explaining 54.46% of the variance. A total of 19 items were eliminated (avoids touch, assistance eating, constipation, flinches when eating, urinary incontinence, ear problems, alopecia, syncope, vomiting, diarrhoea, halitosis, coughing/dyspnoea, greying, masses, weight change, circling, head pressing, seizures, head tilt). Principal Axis Factoring with Promax rotation was performed with the inclusion of all items. The correlation matrix was visually assessed to check the correlation between variables, before extracting factors on the basis of the eigenvalues using Kaiser’s criteria [43], with a minimum requirement of being greater than 1, and the scree plot [44]. The percentage of non-redundant residuals was checked to ensure that less than 50% were greater than 0.5, indicating that the factor model had a good fit [45]. The pattern matrix resulting from the oblique rotation was assessed, retaining items if their loading was greater than 0.4 on one of the factors. The analysis was repeated after each round of exclusions until all items loaded onto at least one of the factors at a value of greater than 0.4 and cross loading was allowed. The internal consistency of the factors was assessed using Cronbach’s α [46], and items were removed if this would improve the reliability of a factor (explained in the results). For simplicity, the factors were then labelled according to the theme identified within the items, guided by the highest loading item. These were used to calculate the overall health score (OHS), equal to the percentage of the overall sum of scores, divided by the total possible score.

Factor Analysis indicated that two factors had a good level of internal consistency, being greater than 0.7 [47] (Factor 1, α = 0.849, Factor 3, α = 0.758). ‘Quidding’ (dropping food from the mouth whilst eating) had to be deleted from factor 2 to improve Cronbach’s α from 0.626 to 0.682. Factor 4 had the lowest measure of internal consistency, α = 0.551. For all the factors, the correlated item-total correlation was over 0.3, indicating that the items correlated adequately with the total score [45]. The final pattern matrix is illustrated in Table 1.

Factor 1 was labelled ‘Musculoskeletal–neurological conditions’, factor 2 was labelled ‘Digestive conditions’, factor 3 was labelled ‘Metabolic conditions’, and factor 4 was labelled ‘Dermatological conditions’. Composite scores were created for each factor based on the mean of the corresponding items, with higher scores indicating a greater health deficit in that system (Table S2 in the Supplementary Information).

A Spearman’s correlation coefficient test indicated that all mean factor scores and the OHS had a positive correlation with the CCAS score (all *p* < 0.001; Bonferroni adjusted alpha = 0.002 for multiple correlations). The correlation was strong (range r = 0.5–1.0) between musculoskeletal–neurological conditions and the CCAS score (r = 0.69), and between the OHS and the CCAS score (r = 0.71). There were moderate (range r = 0.3–0.49) positive correlations between digestive conditions and the CCAS score (r = 0.32) and between metabolic conditions and CCAS scores (rho = 0.36). The correlation was weak (range r = 0.1–0.29) between dermatological conditions and the CCAS (r = 0.27).

An independent sample Kruskal–Wallis test indicated that all composite factor scores were significantly different between the CCAS categories overall. When looking at pairwise comparisons, the factor scores were, on average, significantly higher, comparing normal ageing with both mild and severe cognitive impairment (all *p* values < 0.001) (Supplementary Information, Table S3).

When considering the veterinary diagnosis of musculoskeletal disease, Pearson’s chi-square test (Table 2) indicated a significant but small association between this and the CCAS category (χ^2^ (2) = 28.551, *p* < 0.001, Cramer’s V = 0.201, *p* < 0.001). For the remaining diagnoses, the variable ‘Presence of cognitive impairment’ was used (mild and severe cognitive impairment were considered together). There was a significant but small relationship between the presence of cognitive impairment and the diagnosis of dental disease (χ^2^ (1) = 18.886, *p* < 0.001, Cramer’s V = 0.163, *p* < 0.001). Specifically, the BCS was higher in the presence of signs of cognitive impairment (mean = 3.12, sd = 0.73, skew = −0.24), compared to normal cognition (mean = 3.12, sd = 0.58, skew = 0.13). Similarly, the dental disease score was higher in the presence of signs of cognitive impairment (mean = 0.4, sd = 0.49, skew = 0.4), compared to normal cognition (mean = 0.25, sd = 0.43, skew = 1.16). There was no significant relationship between the remaining diagnoses (gastrointestinal, dermatological, diabetes mellitus, hypothyroidism, hyperadrenocorticism, chronic kidney disease, epilepsy, hepatic disease, cardiovascular disease, and neoplasia) and the presence of cognitive impairment (all *p* = 0.002).

Finally, the regression model (AIC = 4252.68, χ^2^ (5) = 515.57, *p* < 0.001, R_p_^2^ = 0.52) explained 52% of the variance in the CCAS score. Estimates indicated that higher scores in all four factors and in age predicted a higher CCAS score, i.e., more severe signs compatible with CDS (Table 3).

## 4. Discussion

An online survey was performed to investigate the relationship between canine cognitive impairment, as assessed by a validated questionnaire (CCAS) [25], and symptoms of the disease. Positive correlations were found between CCAS scores (a measure of cognitive impairment) and owner-observed behavioural indicators for health, a measure of body condition, and veterinary diagnoses of musculoskeletal and dental disease. A cumulative measure of health, the OHS (Overall Health Score), also had a strong, positive correlation with the CCAS score. These results support the hypotheses that (i) common pathological processes, in the form of factors, would link behavioural signs of disease and that these would be associated with greater cognitive impairment and (ii) that worsening overall health, assessed by the OHS, would be associated with greater cognitive impairment, as would the presence of specific disease states. This suggests that there might be parallelisms between some aspects of physical health and cognitive impairment.

In the current study, the prevalence of owner-reported signs of moderate cognitive impairment in senior dogs was 43%, and the prevalence of severe cognitive impairment was 2%. The most striking finding of this study is that the cognitive status of the dogs (CCAS score) had a strong positive correlation with both the musculoskeletal–neurological factor and the overall health score (OHS) of the current survey. In particular, the musculoskeletal–neurological health was significantly better in the dogs ageing normally (CCAS—Normal Ageing), compared to those with mild and severe decline (CCAS—Mild cognitive impairment and Severe cognitive impairment), with no difference between the two groups of cognitive impairment. This suggests that there is a relationship between the presence of musculoskeletal–neurological conditions and the development of cognitive decline, but not with the severity of cognitive impairment. A diagnosis of musculoskeletal disease by a veterinarian in the preceding year was also found to be significantly associated with the CCAS category, further adding consistency to the findings. However, considering the relatively small number of dogs within the severe cognitive impairment group compared to the other groups, it cannot be excluded that this limited sample size could affect the statistical power to detect differences between the cognitively impaired groups. All of the composite factor scores and the overall health score (OHS) had significant positive correlations with the CCAS score, indicating that higher measures of disease were associated with greater scores on the measure of cognitive impairment. There was a strong correlation of both musculoskeletal–neurological conditions and OHS with cognitive impairment, compared to moderate and weak correlations for the other factors (digestive, metabolic, and dermatological conditions), suggesting that the measures of musculoskeletal and neurological disease might be of the greatest interest when looking further at the relationship between disease and cognitive decline. Age was also significantly positively correlated with the CCAS score, having a fair strength of association, and with the mean age of subjects in each CCAS category rising with increasing severity of cognitive impairment. Age is the main risk factor for CCDS [48], and a positive correlation between cognitive impairment and age mirrors the findings of other published measures of cognitive impairment [8,23].

The associations identified between physical health and results from the CCAS have three potential mechanisms: (1) the disease process may represent a risk factor for the development of cognitive impairment due to its impact on the pathogenesis of CCDS; (2) both the physical health disorder and the cognitive decline may have a common risk factor, such as age, and as a consequence, individuals may be more likely to have both conditions but without any direct causal link between them; (3) the behaviours assessed on the CCAS are not specific to changes seen with cognitive impairment. It should also be noted that the factors identified following analysis of the GHQ describe underlying latent variables representing pathological processes, such as musculoskeletal disease. As the GHQ items consisted of owner-observed behaviours, disease processes resulting in behavioural change readily identifiable by owners would tend to result in factor formation, such as impaired mobility and house soiling [49,50,51].

Additionally, a high prevalence of the underlying disease processes would increase the likelihood of that pathology being represented in the population sampled. It is likely that some pathology leads to discrete behavioural change that occurs independently of other symptoms (for example, brain pathology might only cause one owner-detected symptom, e.g., head tilt) and does not co-vary, meaning that these conditions would not be represented by the factors identified. The four most prevalent disorder groups in dogs [52] in the UK correspond to three of the factors identified in the current study: dental disease (14.10%) and enteropathy (10.43%), corresponding to the factor “digestive conditions”; skin disorders (12.58%) corresponding to the factor “dermatological conditions”; musculoskeletal disorders (8.64%), corresponding to the “musculoskeletal–neurological conditions” factor. This suggests that the behavioural changes identified in the GHQ items were symptomatic of disease states other than CCDS. However, it should be considered that this is a questionnaire-based piece of research, where owners were aware that the general interest was the welfare and cognition of older dogs. CCDS is a diagnosis of exclusion, and chronic pain diseases can mimic the clinical signs, so while the musculoskeletal–neurological diseases might have been actually occurring, owners could have been confounding the signs and giving dogs a higher score due to confirmation bias.

The OHS had a strong, positive correlation with cognitive impairment (CCAS score). There are similar findings in the human literature, where the concept of frailty has been developed, describing a lack of resilience of an individual to disease processes [53]. Frailty indices are cumulative measures of health that are a means of representing this concept. Although the cumulative health measure in the current study was not a frailty index, which has to meet specific requirements, including a wide breadth of indicators [54], it is nevertheless of interest that the OHS was associated with the degree of cognitive impairment, paralleling findings in human medicine. There is epidemiological evidence in humans that increased frailty is related to greater cognitive impairment [55], potentially due to inflammaging [56] and changes in hormonal, vascular, and intracellular systems [55,57]. The upregulation of inflammation that occurs with increasing age is termed ‘inflammaging’ [56,58]. Frailty indices have been developed for use in dogs [35,59], where the inflammaging processes have also been hypothesised [60], and their relationship with cognitive impairment and CCDS would be an important area of future research. Three of the factors identified in this study are commonly associated with inflammatory processes; musculoskeletal–neurological, digestive, and dermatological conditions. Systemic inflammation has been postulated to mediate age-related cognitive decline in humans [61], with increased levels of inflammatory markers associated with higher measures of cognitive impairment [62,63,64]. In the current study, the digestive conditions factor consisted of two items (assistance feeding, decreased appetite) that could be interpreted as representing impairments in the ingestion of food, potentially associated with inflammation, and was moderately, positively correlated with cognitive impairment (CCAS score). An indication that oral health may be contributing to the digestive conditions factor was the finding that a veterinary diagnosis of dental disease was associated with the presence of cognitive decline, whereas there was no significant association between the diagnosis of gastrointestinal disease and the presence of cognitive impairment. The presence of periodontal pathogens has been found to be a risk factor for Alzheimer’s disease in people [65], and it is possible that this might be a mechanism by which, in this study, the digestive factor relates to cognitive decline. Both musculoskeletal–neurological and digestive conditions can also be associated with chronic pain [66], which could account for their correlation with cognitive impairment (CCAS scores) in the current study. Chronic pain is often maladaptive [67] and can persist after the initiating assault has resolved [68]. Pain has been recently found to be associated with behaviour problems in several cases in veterinary medicine [69,70,71,72]. It is often, though not always, associated with inflammation, and has been demonstrated to be linked with cognitive impairment in human patients [73].

The factor relating to musculoskeletal–neurological conditions, which included items pertaining to sensory function, had a strong positive correlation with cognitive impairment. Human age-related hearing loss is significantly associated with both MCI and Alzheimer’s disease [74]. Hearing loss is a recognised risk factor for dementia [75], although it has been proposed that this relationship may be due to a common underlying pathology, which is yet to be elucidated [76]. Changes to olfaction and vision have also been associated with human cognitive decline, impairments that are related to an increased risk of cognitive impairment [77]. In dogs, sensory impairment has been found to be associated with age-related behavioural change [23], while associations between hearing loss and cognitive decline [78], and between hearing loss and cognitive decline [78], have also been found, highlighting the need for sensory testing prior to cognitive testing [26]. In addition, the presence of cataracts has been found to be a marker of reduced lifespan in dogs [79]. While the underlying mechanisms are yet to be fully understood, in relation to DISHAAL signs, it cannot be excluded that dogs with reduced vision and also hearing manifest disorientation, difficulties in navigating their known surroundings, and/or fearful or aggressive responses when approached suddenly.

The metabolic conditions factor was positively moderately correlated with cognitive impairment (CCAS score). Metabolic diseases can lead to a wide range of behavioural changes, including lethargy, dullness, and house soiling [80,81], which can mimic symptoms of CCDS. Additionally, there could even be a causal link between metabolic conditions and cognitive impairment—there are parallels for this finding in human medicine. Diabetes mellitus is associated with an amnestic mild cognitive impairment through vascular disease mechanisms [82], and chronic kidney disease is also associated with cognitive impairment due to altered cytokine production [83]. This latter mechanism relates back to inflammatory processes and highlights how interconnected the body systems are, meaning that they cannot be considered in isolation. There is currently a lack of research on these mechanisms in the veterinary field, so there is no evidence of causative or even mediation effects, but nevertheless, these should be taken into consideration in clinical settings. In addition, being both underweight and overweight was significantly associated with the presence of cognitive impairment, although this association was small. Due to the low number of dogs with severe cognitive impairment, the two categories of cognitive impairment had to be merged to facilitate analysis. As a result, it is not possible to make considerations between the body condition score and the severity of cognitive decline. According to the human literature, the mechanism of this relationship may be a consequence of the chronic, systemic inflammation associated with obesity [84,85] and accompanying pain [86]. The nature of the association between inflammation and malnourishment is not entirely understood, even in humans, although it is linked as part of the malnutrition-inflammation complex syndrome [87].

Finally, only a weak positive correlation was found between the dermatological conditions factor and cognitive impairment (CCAS score). The factor consisted of only two items (pruritus and licking), both of which are readily perceived by owners, which may have contributed to their forming a factor. The human literature reflects this finding, with a study based on care home residents not finding an association between skin disease and cognitive impairment [88]. When considering dermatological conditions, although pruritus and licking are well-accepted to be a response to skin-related discomfort due to dermatopathies [89], less well-investigated is the tendency for dogs to lick and chew at their own bodies in response to other sources of pain [71]. This latter connection is supported by the fact that the item ‘lameness’ had a relatively high cross-loading (below threshold) on the dermatological conditions factor, although it had a higher loading on musculoskeletal–neurological conditions factor, where it was finally included. This finding further supports the hypothesis that licking behaviour may be important clinically as an indicator of non-dermatological discomfort.

Although the associations identified in the current study may be due to disease acting as a risk factor for cognitive decline, being a cross-sectional study, causality could not be determined between the variables studied. In addition, the regression analysis found a main effect of age alone on the CCAS score. Further large-scale, prospective, longitudinal studies are required to investigate the impact of co-variables (such as neuter status, age at neutering, exposure to environmental factors, and physical activity) on the relationship between physical health and cognitive decline and establish the nature of their interaction. There is high variability in the prevalence of CCDS reported in the literature [2,3,5,7,8,9], which may partly be due to differences in study designs and the scales used, but it is also possible that such differences could be indicative of other confounding factors such as undetected medical conditions (e.g., pain). Given the lack of specificity of behavioural change to underlying causes, it is possible that changes due to disease processes other than cognitive impairment, such as pain, could lead to higher scores on the CCAS. Alternatively, there may be many individual mechanisms by which disease states might lead to increased CCAS scores, with the lack of recognition of these associations potentially resulting in increased scores being mistakenly attributed to cognitive decline. As a consequence, if veterinarians suspect a patient may be suffering from CCDS, this study highlights the need to ensure that the patient is screened for other conditions, particularly those that are painful, that might be confusing the clinical picture.

All in all, the interaction between physical health and cognitive decline is clearly a complex one with nuanced links in pathogenesis. The high prevalence of cognitive impairment in senior dogs highlights the importance of their assessment of the condition. Dogs should be regularly screened once they are considered mature (50–75% of their expected lifespan), facilitating early commencement of treatment and management strategies [1], which can help slow down the pathology and improve the quality of life for the dog and owner. Whilst the presence of medical conditions is useful in differentiating between normal ageing and the presence of cognitive impairment, it does not relate to the progression of cognitive impairment from mild to severe. The findings of the current study suggest that dogs who display behaviours suggestive of cognitive impairment should be screened for other medical diseases, especially musculoskeletal pathology, including the presence of pain and sensory decline.

## 5. Conclusions

In conclusion, this study demonstrated that owner-reported behavioural indicators of musculoskeletal, neurological, digestive, metabolic, and dermatological disease are all positively correlated with signs compatible with cognitive impairment. Similar findings have been found for a cumulative measure of health, being over- and under-weight, and having a current diagnosis of musculoskeletal or dental disease made by a veterinarian. Currently, the mechanism underlying these relationships is not determined and might include mediation and/or moderation effects, for example, disease states acting as risk factors for cognitive impairment, the behavioural signs of disease elevating scores on the CCAS, or other common mechanisms (e.g., inflammaging) underlying both. For veterinarians in practice, the findings draw attention to the importance of screening dogs with these health conditions for evidence of cognitive decline to enable early therapeutic intervention, and also to consider the impact of other disease states, particularly pain, on the interpretation of scales used as an aid in the diagnosis of CCDS.

## Figures and Tables

**Table 1:** Pattern matrix of final 4 factors; principal axis factor analysis (bold font = items loading >0.4 for that factor).

	Factor			
	1 Musculoskeletal–Neurological	2 Digestive	3 Metabolic	4 Dermatological
Struggles in/out of the car	**0.741**	−0.028	−0.030	0.160
Tires during exercise	**0.746**	−0.052	−0.045	0.157
Assistance on stairs	**0.680**	0.156	−0.079	−0.034
Activity levels	**0.735**	−0.109	0.022	0.051
Assistance to stand	**0.611**	0.288	−0.036	−0.137
Foot scuffing	**0.630**	0.037	−0.132	0.127
Hearing	**0.599**	−0.088	0.191	−0.166
Sight	**0.470**	−0.038	0.290	−0.101
Play	**0.606**	−0.133	0.157	−0.144
Lameness	**0.598**	−0.070	−0.076	0.316
Faecal incontinence	**0.412**	0.207	−0.060	−0.373
Assistance feeding	−0.070	**0.880**	0.032	0.014
Decreased appetite	−0.032	**0.831**	0.043	0.120
Polyuria	−0.035	0.033	**0.872**	0.071
Polydipsia	0.032	0.046	**0.820**	0.075
Pruritic	0.072	0.005	0.008	**0.759**
Licks body	0.045	0.140	0.129	**0.702**
Eigenvalues	5.09	1.55	1.35	1.27
% of variance	29.93	9.12	7.97	7.44
α	0.85	0.68	0.76	0.55

Deleted items: avoids touch, assistance eating, constipation, flinches when eating, urinary incontinence, ear problems, alopecia, syncope, vomiting, diarrhoea, halitosis, coughing/dyspnoea, greying, masses, weight change, circling, head pressing, seizures, head tilt.

**Table 2:** Analyses of the relationship between the variables breed, body condition and veterinary diagnosis of disease, and measures of cognitive impairment (adjusted alpha for multiple comparisons = 0.002, significant *p* values in bold).

	n	df	Pearson Chi-Square	*p*-Value	Cramer’s V
Cognitive state: normal vs. impaired					
Body condition	804	2	21.25	**<0.001**	0.163
Dental disease	710	1	18.87	**<0.001**	0.163
Gastrointestinal disease	710	1	2.33	0.127	0.057
Dermatological disease	710	1	0.855	0.355	0.035
Hypothyroidism	710	1	0.143	0.705	0.014
Hyperadrenocorticism	710	1	1.657	0.198	0.048
Chronic kidney disease	710	1	2.831	0.092	0.063
Epilepsy	710	1	0.011	0.918	0.04
Hepatic disease	710	1	2.019	0.155	0.053
Cardiovascular disease	710	1	4.183	0.041	0.077
Cancer	710	1	0.270	0.603	0.020
**Cognitive state: normal vs. mild vs. severe impairment**					
Musculoskeletal disease	710	2	28.55	**<0.001**	0.201
Breed category	804	2	0.537	0.764	0.026

**Table 3:** Regression model including the four principal axis factors and age. Estimates are reported with standard errors.

Predictor	Estimate (±S.E.)	*p*
Factor 1 (Musculoskeletal–neurological)	1.84 (0.15)	0.001
Factor 2 (Digestive)	0.25 (0.12)	0.040
Factor 3 (Metabolic)	0.51 (0.12)	0.001
Factor 4 (Dermatological)	0.47 (0.08)	0.001
Age (years)	0.29 (0.03)	0.001

## Data Availability

Data are available upon request from the authors.

## Supplementary Materials

The following supporting information can be downloaded at: https://www.mdpi.com/article/10.3390/ani13132203/s1, List of questions related to the signs of medical conditions, List of questions about veterinary diagnoses of medical conditions, Tables S1–S3: Table S1. Correlation matrix of signs of medical conditions. Table S2. The descriptive statistics for the factors extracted from the GHQ. Table S3. Independent samples Kruskal–Wallis test. Degree of cognitive impairment and composite factor scores.
