# Supplementary material for: The Relationship between Signs of Medical Conditions and Cognitive Decline in Senior Dogs
Source: Animals (Basel). 2023 Jul 5;13(13):2203. doi: 10.3390/ani13132203 (PMC10340034; doi:10.3390/ani13132203)
Supplement: Supplementary file 1 [file animals-13-02203-s001.zip › animals-2425738-supplementary.pdf]

<u>Supplementary information for the paper "Relationship between signs of medical conditions and cognitive decline in senior dogs"</u>

List of questions relative to the signs of medical conditions:

Please indicate how often your dog shows the following behaviours using the scoring system:

0 = Never, 1 = Once a month, 2 = Once a week, 3 = Almost every day

1. Needs assistance to stand up
2. Requires assistance to climb the stairs
3. Limps when moving
4. Tires quickly during exercise compared with previously
5. Struggles to get in and/or out of the car
6. Avoids being touched by yourself when it did not used to
7. Licks one part of the body repetitively when it did not used to
8. Has a decreased appetite
9. Needs assistance eating, for example hand feeding
10. Suffers from bouts of vomiting
11. Suffers from bouts of diarrhoea
12. Suffers from constipation
13. Suffers from faecal incontinence (passing faeces without being aware of it), note this does not refer to problems with house training
14. Has an unpleasant mouth odour
15. Drops food from their mouth when eating
16. Flinches when eating

17. Scratches or nibbles itself repeatedly
18. Has urinary incontinence (passing urine without being aware of it), note this does not refer to problems with house training or when dogs urinate when excited/submissive
19. Has bouts of circling repeatedly in one direction
20. Presses its head against surfaces
21. Scuffs its feet as it walks along
22. Has bouts of coughing or struggling to breath
23. Has episodes of fainting (losing consciousness)

Using the chart below (N.B. WSAVA 9 point Body Condition Score) to answer the following questions

0 = no weight gained; 1 = a little weight gain, their BCS has increased by 1 point; 2 = moderate weight gain, their BCS has increased by 2-3 points; a lot of weight gained, their BCS has increased by 4 or more points

24. Over the last six months how much has your dog's weight INCREASED (without changes to diet or exercise regime)?

0 = no weight lost; 1 = a little weight loss, their BCS has decreased by 1 point; 2 = moderate weight loss, their BCS has decreased by 2-3 points; a lot of weight lost, their BCS has decreased by 4 or more points

25. Over the last six months how much has your dog's weight DECREASED (without changes to diet or exercise regime)?

Please indicate to what degree your dog experiences the following conditions using the scoring system:

26. Hearing impairment (*0 = He/she can hear perfectly, 1 = He/she can hear most sounds, 2 = He/she can hear only loud sounds, 3 = He/she is completely deaf*)
27. Visual impairment (*0 = He/she can see perfectly, 1 = He/she can see but vision is somewhat limited (occasionally bumps into things or has problems identifying objects), 2 = His/her vision is extremely limited (they can only see shadows and exaggerated movements), 3 – He/she is completely blind*)

28. How frequently does your dog show playful behaviour? (*0 = Plays multiple times a day, 1 = Plays every day or almost every day, 2 = Plays 1-2 times a week, 3 = Plays once a month or less*)
29. How active is your dog? (*0 = Runs every time that he/she gets, 1 = He likes to have a good run at least once a day, 2 = He runs sometimes, but stops frequently or is very tired afterwards, 3 = He very rarely runs around or never does*)
30. How much is your dog's fur greying? (*0 = No new grey hairs OR Not Applicable (the coat colour is already grey/white, 1 = A few new grey hairs present, 2 = Coat colour of some areas of the body starting to change due to new grey hairs, 3 = Areas of the body becoming very grey, e.g. the muzzle*)
31. Has your dog suffered from any ear problems over the last six months (either a new problem or a flare up of ongoing issues)? *0 = Not at all, 1 = Once a month, 2 = Once a week, 3 = Almost every day*
32. Has your dog suffered from areas of hair loss over the last six months? *0 = Not at all, 1 = Mild thinning of hair in areas, 2 = Marked thinning of hair in areas which is noticeable from a distance, 3 = Some areas completely hairless*
33. Does your dog have lumps on his/her body or in his/her mouth? *0 = Not at all, 1 = A couple of small lumps present but not impacting on your dog i.e. your dog doesn't seem to notice them, 2 = Several lumps present and/or lumps are having a minor impact on your dog e.g. your dog occasionally licks them, 3 = Many lumps are present and/or lumps are significantly impacting your dog e.g. are painful*
34. Does your dog urinate more than it used to? *0 = Not at all, 1 = Urinating a bit more than normal, 2 = Urinating about twice as much as normal, 3 = Needing to urinate extremely frequently e.g. every hour*
35. Does your dog drink more than it used to? *0 = Not at all, 1 = Drinking a bit more than normal, 2 = Drinking about twice as much as normal, 3 = Needing to drink extremely frequently e.g. every half an hour*
36. Has your dog experienced seizures in the last six months? *0 = Not at all, 1 = 1-2 seizures which resolved on their own, 2 = Several seizures which resolved on their own, 3 = Very frequent seizure which required medication to bring them to an end*
37. Over the last six months how often have been periods of at least 24 hours when your dog has held their head permanently tilted to one side? *0 = Never, 1 = On one occasion, 2 = On more than one occasion, 3 = Continuously.*

List of questions about veterinary diagnoses of medical conditions

Has your pet suffered from any of the following conditions (with an official diagnosis provided by a vet) over the last year?

0 = no, 1 = yes

1. Musculoskeletal conditions, for example osteoarthritis?

2. A chronic digestive disease, for example inflammatory bowel disease?
3. Poor oral health, for example advised or carried out tartar removal or dental extractions?
4. Skin disease, for example skin allergies
5. Diabetes mellitus
6. Hypothyroidism
7. Hyperadrenocorticism (also referred to as Cushing’s Disease)
8. Chronic kidney disease
9. Epilepsy
10. Heart disease
11. Liver disease
12. Cancer

Table S1. Correlation matrix of signs of medical conditions.

	Struggles in/out car	Tires during exercise	Assistance on stairs	Activity levels	Assistance to stand	Foot scuffing	Hearing	Sight	Play	Lame-ness	Faecal incontinence	Assistance feeding	Decreased appetite	Polyuria	Polydipsia	Pruritic	Licks body
Struggles in/out car	1.000	0.536	0.497	0.428	0.433	0.417	0.393	0.297	0.310	0.446	0.249	0.198	0.231	0.175	0.227	0.165	0.198

Tires during exercise		1.000	0.443	0.495	0.397	0.419	0.306	0.279	0.360	0.467	0.213	0.182	0.259	0.164	0.223	0.146	0.144
Assistance on stairs			1.000	0.405	0.602	0.349	0.355	0.314	0.306	0.371	0.278	0.253	0.261	0.140	0.186	0.071	0.142
Activity levels				1.000	0.358	0.360	0.342	0.321	0.560	0.323	0.168	0.204	0.217	0.180	0.143	0.138	0.138
Assistance to stand					1.000	0.461	0.334	0.288	0.280	0.359	0.417	0.304	0.302	0.183	0.242	0.022	0.110
Foot scuffing						1.000	0.281	0.193	0.214	0.341	0.247	0.179	0.171	0.138	0.143	0.133	0.157
Hearing							1.000	0.488	0.361	0.179	0.233	0.161	0.182	0.175	0.172	0.078	0.090
Sight								1.000	0.299	0.181	0.228	0.172	0.192	0.218	0.198	0.115	0.140
Play									1.000	0.188	0.168	0.159	0.187	0.152	0.179	-0.004	0.108
Lameness										1.000	0.139	0.114	0.170	0.128	0.221	0.163	0.196

Faecal incontinence											1.000	0.187	0.117	0.039	0.131	-0.053	-0.020
Assistance feeding												1.000	0.554	0.169	0.154	0.088	0.111
Decreased appetite													1.000	0.160	0.183	0.077	0.214
Polyuria														1.000	0.610	0.088	0.160
Polydipsia															1.000	0.089	0.179
Pruritic																1.000	0.384
Licks body																	1.000

Table S2. The descriptive statistics for the factors extracted from the GHQ

**Factor**	**Label**	**Number of items**	**Mean**	**SD**	**Skewness**	**Kurtosis**

1	Musculoskeletal-neurological	11	0.88	0.62	0.63	0.53
2	Digestive	2	0.25	0.59	2.75	7.31
3	Metabolic	2	0.43	0.59	1.53	2.13
4	Dermatological	2	0.58	0.82	1.36	1.00

Table S3. Independent samples Kruskal-Wallis test; Degree of cognitive impairment and composite factor scores**.** Mean score and p values are reported.

			**Mean factor score**		
	**Chi squared (df)**	***P* – value**	**NA**	**MCI**	**MSCI**
Musculoskeletal-neurological conditions	240 (2)	<0.001	0.58	1.27	1.77
Digestive conditions	67.48(2)	<0.001	0.1	0.41	1.15
Metabolic conditions	64.00(2)	<0.001	0.3	0.61	0.88

Dermatological conditions	42.03(2)	<0.001	0.4	0.82	1.38

N.B. Adjusted alpha level for multiple comparisons = 0.002; NA = Normal ageing, MCI = Mild cognitive impairment, SCI = Severe cognitive impairment
